# Multidrug resistance among *Escherichia coli* and *Klebsiella pneumoniae* carried in the gut of out-patients from pastoralist communities of Kasese district, Uganda

**DOI:** 10.1371/journal.pone.0200093

**Published:** 2018-07-17

**Authors:** Iramiot Jacob Stanley, Henry Kajumbula, Joel Bazira, Catherine Kansiime, Innocent B. Rwego, Benon B. Asiimwe

**Affiliations:** 1 Department of Medical Microbiology, College of Health Sciences, Makerere University, Kampala, Uganda; 2 Department of Microbiology and Immunology, Faculty of Health Sciences, Busitema University, Kampala, Uganda; 3 Department of Microbiology, Faculty of Medicine, Mbarara University of Science and Technology, Kampala, Uganda; 4 One Health Central and Eastern Africa (OHCEA) network, School of Public Health, Makerere University, Kampala, Uganda; 5 Department of Biosecurity, Ecosystems and Veterinary Public Health, College of Veterinary Medicine, Animal Resources and Biosecurity, Makerere University Kampala, Uganda; 6 Ecosystem Health Division, College of Veterinary Medicine, University of Minnesota, St. Paul, MN, United States of America; Zhejiang University, CHINA

## Abstract

**Background:**

Antimicrobial resistance is a worldwide public health emergency that requires urgent attention. Most of the effort to prevent this coming catastrophe is occurring in high income countries and we do not know the extent of the problem in low and middle-income countries, largely because of low laboratory capacity coupled with lack of effective surveillance systems. We aimed at establishing the magnitude of antimicrobial resistance among *Escherichia coli* and *Klebsiella pneumoniae* carried in the gut of out-patients from pastoralist communities of rural Western Uganda.

**Methods:**

A cross-sectional study was carried out among pastoralists living in and around the Queen Elizabeth Protected Area (QEPA). Stool samples were collected from individuals from pastoralist communities who presented to the health facilities with fever and/or diarrhea without malaria and delivered to the microbiology laboratory of College of Health Sciences-Makerere University for processing, culture and drug susceptibility testing.

**Results:**

A total of 300 participants fulfilling the inclusion criteria were recruited into the study. Three hundred stool samples were collected, with 209 yielding organisms of interest. Out of 209 stool samples that were positive, 181 (89%) grew *E*. *coli*, 23 (11%) grew *K*. *pneumonia*e and five grew Shigella. Generally, high antibiotic resistance patterns were detected among *E*. *coli* and *K*. *pneumoniae* isolated. High resistance against cotrimoxazole 74%, ampicillin 67%, amoxicillin/clavulanate 37%, and ciprofloxacin 31% was observed among the *E*. *coli*. In *K*. *pneumoniae*, cotrimoxazole 68% and amoxicillin/clavulanate 46%, were the most resisted antimicrobials. Additionally, 57% and 82% of the *E*. *coli* and *K*. *pneumoniae* respectively were resistant to at least three classes of the antimicrobials tested. Resistance to carbapenems was not detected among *K*. *pneumoniae* and only 0.6% of the *E*. *coli* were resistant to carbapenems. Isolates producing ESBLs comprised 12% and 23% of *E*. *coli* and *K*. *pneumoniae* respectively.

**Conclusion:**

We demonstrated high antimicrobial resistance, including multidrug resistance, among *E*. *coli* and *K*. *pneumoniae* isolates from pastoralist out-patients. We recommend a One Health approach to establish the sources and drivers of this problem to inform public health.

## Introduction

Antimicrobial resistance is a worldwide public health emergency that requires urgent global attention [1], [2]. Intensive usage of antimicrobials for food animals may cause problems in the treatment of infections by selecting for resistance among bacterial pathogens from animals and humans [3], [4]. Transmission of resistant bacteria from animals to humans may occur via the food chain, environment or by direct interaction with animals and may result in resistant infections. Although *E*. *coli* and *K*. *pneumoniae* are commensals, they are also common pathogens in urinary tract infections and sepsis. Exposure of such bacteria to antimicrobials increases the prevalence of carriage of drug resistant bacteria in animals and humans and this can result in longer duration of hospitalization and increased morbidity and mortality in the human population [5]. Multi-drug resistant infections have been reported to account for mortality of at least 23,000 people in the United states annually [6] while the magnitude of the problem in low and middle-income countries(LMIC) is projected to be three times higher [7]. The global emergency and spread of multi-drug resistant Enterobacteriaceae especially CTX-M type ESBLs and strains producing carbapenemases such as KPC and NDM has made bacteria become increasingly untreatable especially in LMIC [8].

Abuse of antibiotics has resulted into the fast-growing problem of antimicrobial resistance on far, in health care settings [9, 10] and the community [11] [12] [13, 14] Previous studies in Uganda report inappropriate usage of antimicrobials in animal husbandry as a major contributor to the emergence of antimicrobial resistance among microbes [15]. Additionally, 40% of the individuals who visit a health- care facility in Uganda are treated with antibiotics [4] [16]. These antibiotics are mostly given over the counter in drug shops and community pharmacies in sub-therapeutic doses. This may result in multi-drug resistance among pathogenic and non-pathogenic bacteria in humans, which may then be transmitted to the environment and animals as the cycle continues. Queen Elizabeth Conservation area in Kasese district, Uganda, has pastoralists communities with domestic animals grazing inside and adjacent to the park [17], creating a porous interface for transmission of infectious agents and antimicrobial resistance. We aimed at establishing the status of antibiotic resistance among indicator bacteria *E*. *Coli* and *K*. *pneumoniae* isolated from pastoralist out-patients presenting at Health Centres in and around Queen Elizabeth Conservation Area, rural Western Uganda.

## Materials and methods

### Study area and setting

**Fig 1:**
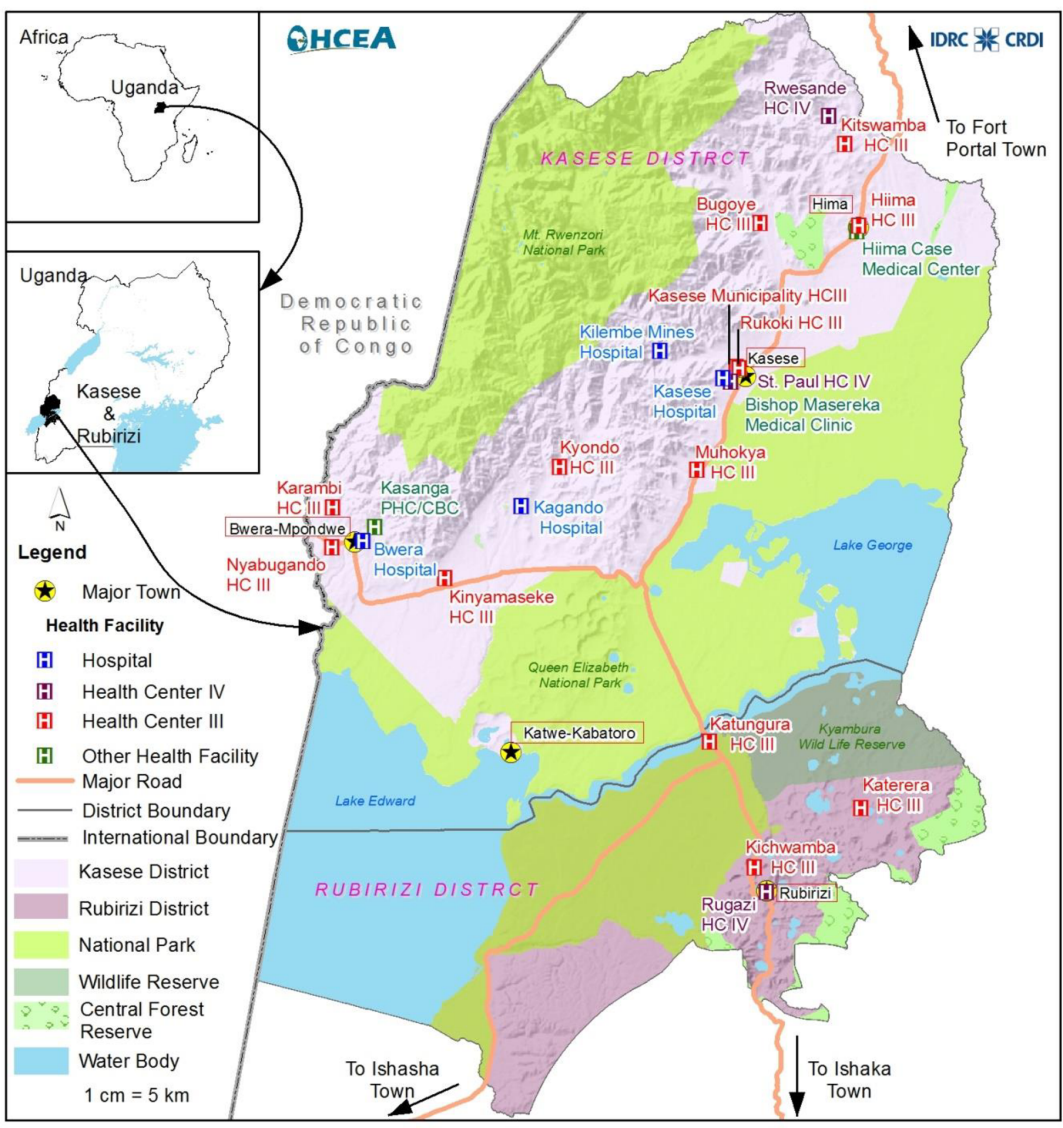
Map of Kasese district showing the distribution of health facilities.

A cross-sectional study was carried out among pastoralists living in and around the Queen Elizabeth Protected Area (QEPA). The QEPA lies astride the equator along the latitudes of 0° 39' 36" North, 30° 16' 30" East. QEPA is located in western part of Uganda on the floor of the western arm of the East African Rift Valley. QEPA forms part of an extensive trans boundary ecosystem that includes Kibale National Park to the northeast, Rwenzori Mountains National Park to the northwest and is also contiguous with Virunga National Park in the Democratic Republic of Congo. The northern area has been occupied by pastoralists since the 1920s (Fig 1). The pastoralists live adjacent to the park and illegally graze inside the park, creating a very porous wildlife-livestock-human interface and a high disease burden for both humans and livestock [17].

### Study population and sampling

Participants were recruited at four health facilities adjacent to the QEPA (Fig 1): Bwera hospital, Kagando hospital, Katwe Health Centre III and Hima Health Centre III. Bwera hospital and Kagando hospital are the major referral centres for patients from the pastoral community health centres whereas Katwe Health Centre III and Hima Health Centre III are health facilities within the pastoralist communities hence their suitability for the study. The inclusion criteria were participants who reared animals, presented with a fever and/or diarrhea but without malaria. Malaria was ruled out by microscopy. The inclusion started in September 2017 and ended in December 2017. A total of 300 stool and 300 blood samples were obtained from 300 participants who presented to the health facilities fulfilling the inclusion criteria.

### Sample collection and transportation

**Fig 2:**
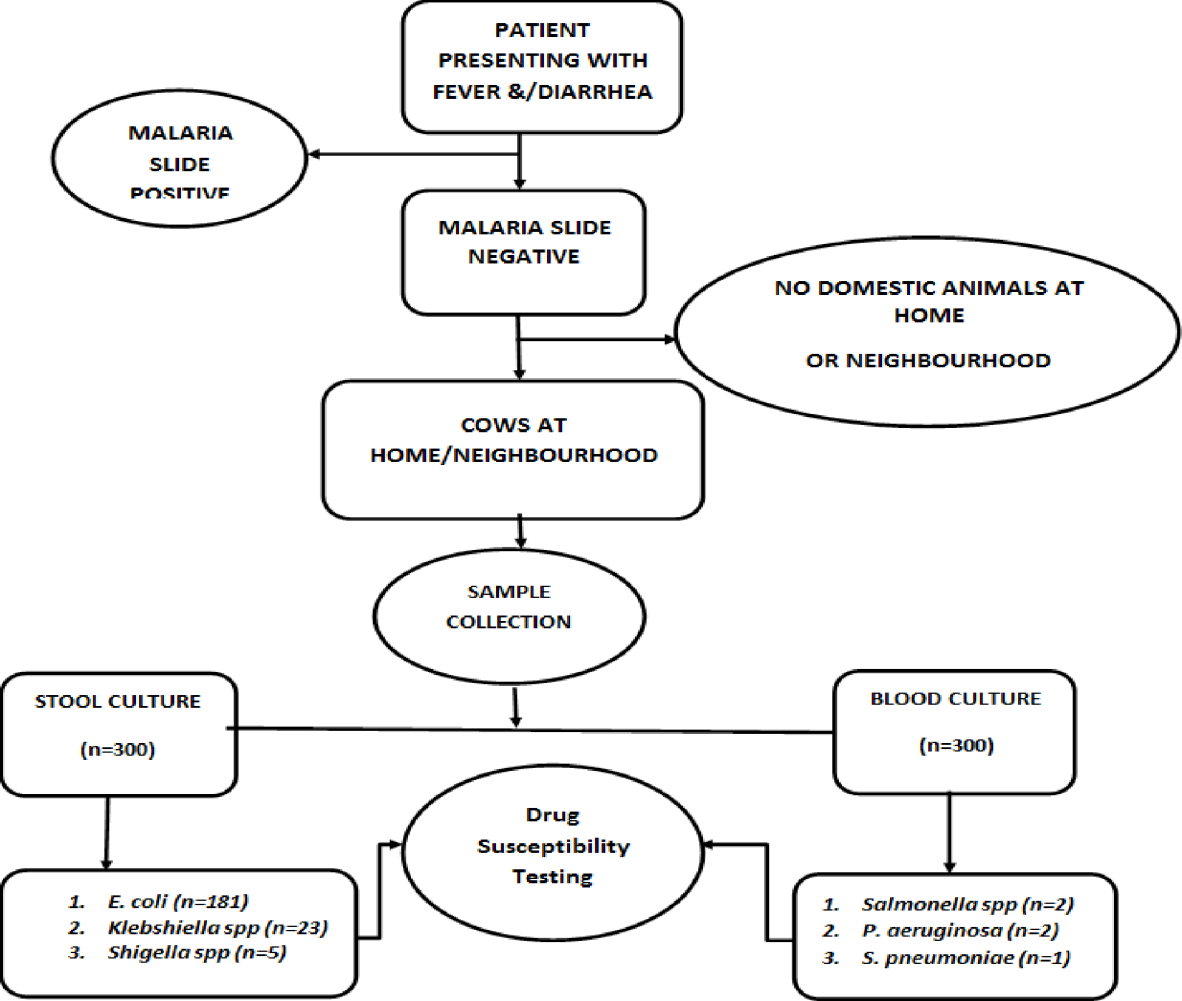
Study profile.

Stool samples were collected into sterile leak proof stool containers with screw caps and kept at 4°C for transportation to the laboratory within 24 hours of collection in Carry Blaire transport medium (Fig 2). Sample processing, culture and subsequent tests were performed at the Clinical Microbiology Laboratory of department of medical microbiology of College of Health Sciences-Makerere university.

### Culture and isolation

All specimens collected were delivered and processed as per standard operating procedures of the microbiology laboratory of Makerere University College of Health Sciences. This laboratory currently participates in proficiency testing and is under evaluation for College of American Pathologists (CAP) certification. Stool samples were first emulsified in sterile normal saline before inoculation to MacCkonkey agar and then incubated at 37^°^C for 24 hours. Lactose fermenting bacterial colonies with colonial morphology suggestive of *E*. *coli* and *K*. *pneumoniae* were isolated and further identified using the Phoenix automated identification system.

### Bacterial identification and susceptibility testing

Bacterial identification and antibiotic susceptibility testing was done using the BD Phoenix 100 automated identification system (Becton Dickinson, Oxford, UK) [18]. The phoenix system has an advantage of combining identification, antimicrobial susceptibility testing and growth and fluorescent controls. The identification (ID) portion of the phoenix panel exploits a series of conventional, chromogenic and fluorogenic biochemical tests to arrive at the identity of the organism. The phoenix also identified ESBL phenotypes. ESBLs are gram negative bacteria producing beta lactamase enzyme therefore capable of breaking down penicillins and cephalosporins and render them ineffective. Following initial culturing, pure cultures were grown from single colonies [19]. One pure colony from each sample was used for identification and antibiotic susceptibility testing (ID/AST). Conventional identification involved use of biochemical tests like indole, methyl-red, voges-proskaur and Simon’s citrate. Only results of automated ID/AST were analyzed for this study. Common drugs used for treatment of suspected Gram negative bacterial infection in Uganda were tested, plus other reserve drugs for Gram negative bacteria commercially available in our setting. A total of 15 drugs used included ampicillin 10μg, amoxicillin/clavunate 20/10μg, cefazolin 30μg, cefuroxime 30μg, ceftazidime 30μg, Ceftriaxone 30μg, Cefepime 30μg, Erteapenem 10μg, Imipenem 10μg, Gentamycin10μg, Ciprofloxacin 5μg, Levofloxacin 5μg, Tetracycline 30μg, Nitrofurantoin 10μg and Cotrimoxazole 1.25/ 23.75μg. Multidrug resistance was defined as one isolate being resistant to three or more classes of antibiotics tested [20–22]. For the case of *K*. *pneumoniae*, this definition applies with exception of ampicillin since Klebsiella is known to be intrinsically resistant to ampicillin. For the case of ESBLs-MDR, the above definition was used with exception of penicillins and cephalosporins. The AST results obtained were interpreted using the CLSI break points. The *E*. *coli* isolate was also subcultured on sorbitol MacConkey to screen for possible zoonotic origin.

### Data management

Data collected from the study was entered, cleaned using Microsoft excel and imported to Stata version 14 for analysis. The primary outcome was multi-drug resistance and the secondary outcome was ESBL. Data was analyzed using descriptive statistics, frequencies and bivariate analyses (cross-tabulations). Associations were tested using Pearson’s Chi square. A significant level was set at p = 0.05.

### Data quality control

Laboratory procedures were performed by laboratory scientists under close supervision of a clinical microbiologist to ensure quality results are obtained. Data was double entered into Microsoft excel for accuracy and reliability. ATCC 259922 *E*. *coli* was used as a control strain for quality assurance during isolation and drug susceptibility testing.

### Ethics approval and consent to participate

The study was approved by the Makerere University School of Biomedical Sciences Higher Degrees Research and Ethics Committee (SBS-HDREC) and written informed consent was obtained from participants.

## Results and discussions

### Results

#### Socio-demographic characteristics of the study participants

Three hundred (300) participants presenting with fever and/or diarrhea took part in this study. The mean age of the respondents was 21.7 -/+ 14.5 years. The youngest participant was 1 year and the oldest 70 years. Participants below 20 years constituted the majority, (n = 174; 58%) while those above 60 years constituted the minority, (n = 7; 2.3%). Of the 300 participants recruited, 241 presented with fever while 59 reported with diarrhea. Demographic characteristics such as age group, health facility or sex were not associated with MDR or ESBL production “Table 1”. There was no significant difference in ESBL prevalence between the patient group that presented with fever, and the group that presented with diarrhea.

**Table 1:** Socio demographic characteristics and distribution of drug resistance.

	MDR n (%)	OR(95% CI)	P-value	ESBL n (%)	OR (95% CI)	P-value
**Sex**						
**Male**	48 (42)	1		11 (39)	1	
**Female**	67 (58)	1.13(0.652–1.992)	0.3	17 (61)	1.2(0.532–2.715)	0.658
**Age group (years) **						
**1–10**	30 (26)	1		5 (18)	1	
**11–20**	31 (27)	0.56(0.263–1.209)	0.141	6 (21)	0.89(0.255–3.107)	0.855
**21–30**	21 (18)	1.05(0.419–2.632)	0.917	7 (25)	2.32(0.666–8.055)	0.187
**31–40**	16 (14)	0.87(0.333–2.290)	0.782	6 (21)	2.46(0.672–8.984)	0.174
**Above 40**	17 (15)	0.68(0.274–1.685)	0.405	4 (14)	1.23(0.304–0.497)	0.773
**Health facility**						
**Bwera**	36 (31)	1		7 (25)	1	
**Kagando**	14 (12)	0.58(0.224–1.518)	0.3	5 (18)	1.6(0.455–5.623)	0.465
**Katwe**	22 (19)	0.44(0.197–0.984)	0.18	4 (14)	0.62(0.171–2.283)	0.477
**Hima**	43 (37)	0.63(0.307–1.303)	0.23	12 (43)	1.24(0.454–3.386)	0.675

MDR = Multi-drug resistant, ESBL = Extended Spectrum Beta lactamase, OR = Odds ratio

#### Antimicrobial resistance

Out of the 209 stool samples that were positive on culture, 87% (n = 181) grew *E*. *coli*, 23 (11%) were *K*. *pneumonia*e and five were Shigella. Shigella species were not analyzed because we only had few isolates (Fig 2). We screened for zoonotic *E*. *coli* using sorbitol MacConkey and we found that 16% of the *E*. *coli* were categorized as zoonotic using this method. Resistance against cotrimoxazole was 74%, ampicillin 67%, amoxicillin/clavulanate 37%, and ciprofloxacin 31% was noted among the *E*. *coli* whereas among *K*. *pneumoniae*; cotrimoxazole 68%, amoxicillin/clavulanate 46%, were the most resisted. MDR was reported among 122 (59.8%) of the total Isolates whereas ESBL was reported in 28 (13.7%). Additionally, 57% and 82% of the *E*. *coli* and *K*. *pneumoniae* respectively were resistant to at least three classes of antibiotics. None of the *K*. *pneumoniae* was resistant to carbapenems and only 0.6% of the *E*. *coli* was resistant to carbapenems “Table 2”.

**Table 2:** Proportion of *E*. *coli and K*. *pneumoniae* isolates resistant to drugs classes and individual drugs.

Antimicrobial categories	Drugs	*E*. *coli* (n = 181)		*K*. *pneumoniae* (n = 22)		Total
		N (%) by drug	N (%) by category	N (%) by drug	% by category	
**Penicillins**	**Ampicillin**	**121(66.8)**	**121(66.8)**	**IR**	**IR**	**121(66.8)**
**β-lactamase inhibitors**	** Amoxicillin-clavulanic acid**	** 66 (36.5)**	** 66(36.5)**	**11 (47.8)**	** 11(47.8)**	** 77(37.7)**
**1st generation cephalosporins**	**Cefazolin**	**75 (41.4)**	**75(41.4)**	**11 (47.8)**	**11(47.8)**	**86(42.2)**
**2nd generation cephalosporins**	**Cefuroxime**	**38 (21)**	**38(21.0)**	**8 (34.8)**	**8(34.8)**	**46(22.5)**
**Extended Spectrum cephalosporins; 3rd and 4th generation cephalosporins**	**Ceftazidime**	**22 (12.2)**	**21(11.8)**	**5 (21.7)**	**6(24.6)**	**27(13.2)**
	**Ceftriaxone**	**21 (11.6)**		**6 (26.1)**		**27(13.2)**
	**Cefepime**	**21 (11.6)**		**6 (26.1)**		**27(13.2)**
**Quinolones/ Fluoroquinolones**	**Ciprofloxacin**	**27 (14.9)**	**25(13.8)**	**3 (13.0)**	**3(10.9)**	**30(14.7)**
	**Levofloxacin**	**23 (12.7)**		**2 (8.7)**		**25(12.3)**
**Aminoglycosides**	**Gentamycin**	**11 (6.1)**	**11(6.1)**	**3 (13.0)**	**3(13.0)**	**14 (6.9)**
**Tetracyclines**	**Tetracycline**	**64 (35.4)**	**64(35.4)**	**12 (52.2)**	**12(52.2)**	**76(37.3)**
**Nitrofurantoins**	**Nitrofurantoin**	**33 (18.2)**	**33(18.2)**	**15 (65.2)**	**15(65.2)**	**48(23.5)**
**Carbapenems**	**Imipenem**	**1 (0.6)**	**1 (0.6)**	**1 (4.3)**	**1(4.3)**	**2 (1.0)**
	**Erteapenem**	**1 (0.6)**		**0 (0)**		**1 (0.5)**
**Folate pathway inhibitors**	**Cotrimoxazole**	**133(73.5)**	**133(73.5)**	**15 (65.2)**	**15(65.2)**	**148(72.5)**

IR = inherently resistant; % = percentage.

#### Resistance to different antibiotic classes

The prevalence of resistance of the isolates to Penicillins like ampicillin among the *E*. *coli* was 66.8% and 51.6% resistance to β-lactamase inhibitors such as amoxicillin-clavulanic acid. Also 11.8% of *E*. *coli* and 24.6% of *K*. *pnuemoniae* were resistant to the 3^rd^ and 4^th^ generation cephalosporins “Table 2”. All non-ESBL isolates were sensitive to Carbapenems while 3.6% of the ESBL were resistant to carbapenems. The 28 ESBL isolates were significantly more resistant to Quinolones/Fluoroquinolones, Aminoglycosides, Tetracyclines, Nitrofurantoins and Folate pathway inhibitors than the non ESBL producers, p<0.05 “Table 3”.

**Table 3:** Resistance pattern of ESBL and Non-ESBL isolates to different drug categories.

Antibiotic category	NON ESBL (n = 176)	ESBL (n = 28)	Total (n = 204)	*P-value*
Quinolones/Fluoroquinolones	13 (7.4%)	17 (60.7%)	30 (14.7%)	*P<0*.*05*
Aminoglycosides	2 (1.1%)	12 (42.9%)	14 (6.9%)	*P<0*.*05*
Tetracyclines	49 (27.8%)	27 (96.4%)	76 (37.3%)	*P<0*.*05*
Nitrofurantoins	34 (19.3%)	14 (50%)	48 (23.5%)	*P<0*.*05*
Carbapenems	0 (0%)	1 (3.6%)	1 (0.5%)	*P<0*.*05*
Folate pathway inhibitors	122 (69.3%)	26 (92.9%)	148 (72.5%)	*P<0*.*05*

### Discussion

#### Prevalence and antibiotic resistance

Out of 300 stool samples cultured, 209(69.7%) grew Gram negative bacteria of interest. A related study showed a higher recovery rate (87%) for *E*. *coli* and *K*. *pnuemoniae* combined in Kampala and rural districts of Uganda [23]. The same study revealed 97% prevalence *E*. *coli* and 16 3% of *K*. *pneumonia*e. This discrepancy with other studies may be due to different identification systems employed. *E*. *coli* was the most frequently isolated bacterium in this study (60.3%) and this makes its role as a conduit of drug resistance to pathogenic organism important to study. Another organism with the potential of transmission from animals to humans isolated was *K*. *pnuemoniae* (7.7%). Bacteria isolated from blood and Shigella from stool were not included for analysis in this study due to their small proportions, but the results were given to the clinicians for patient management (Fig 2). This study categorized 16% of *E*. *coli* as zoonotic using sorbitol MacConkey. Whereas growth on sorbital MacConkey is not confirmatory of zoonotic *E*. *coli*, our inclusion criteria involving participants with animals or having animals at the neighborhood may indicate a high likelihood of transmission of antimicrobial resistance from animals to humans. However, our results only remain speculative.

Overall, high resistance patterns were detected among our isolates. Our study demonstrates a high prevalence of resistance to cotrimoxazole (74%, 65%) among *E*. *coli* and *K*. *pneumoniae* respectively. The high prevalence to cotrimoxazole is probably due to the wide use of the drug for prophylaxis in HIV care especially that the pastoralists lived close to the fishing villages where the HIV prevalence is usually high. Studies in Uganda and the rest of East African region have reported similar findings [24, 25], [26]. Like in related studies [23], [27], [28], resistance to ampicillin in this study was high (67%) and similarly cefazolin (41%), amoxicillin/clavulanate (37%), tetracycline (35%) and ciprofloxacin (31%) among the *E*. *coli*. Among *K*. *pneumoniae*; cotrimoxazole (68%), nitrofurantoin (64%), tetracycline (50%), amoxicillin/clavulanate (46%) and cefazolin (46%) were the most resisted. Similar trends were reported by other studies [27], [28] indicating that the burden of antimicrobial resistance may be uniformly distributed across East Africa.

#### Resistance to different drug categories

The resistance rates of the isolates to Penicillins and β-lactamase inhibitors tested in this study, i.e ampicillin and amoxicillin-clavulanic acid were 66.8% and 51.6% among *E*. *coli*. Resistance to amoxicillin-clavulanic acid, a beta lactam inhibitor among *K*. *pneumoniae* was 47.8%. In a study in Tanzania, at least 43.5% of isolates were reported as resistant to 3^rd^ generation cephalosporins [27]. This is similar to the trends observed in this study with 11.8% of *E*. *coli* and 24% of K. pneumoniae showing resistance to 3^rd^ and 4^th^ generation cephalosporins. All non-ESBL producing isolates were susceptible to Carbapenems while ESBL resistance to carbapenems was 3.6%. In neighboring Kenya, none of the 912 gram negative bacteria tested was resistant to carbapenems implying that carbapenems were still effective for the treatment of illnesses caused by these organisms [29]. The 28 ESBL isolates were significantly more resistant to Quinolones/Fluoroquinolones, Aminoglycosides, Tetracyclines, Nitrofurantoins and Folate pathway inhibitors than the non-ESBL isolates (*P<0*.*05*). Comparable results were reported in Tanzania [14] where ESBL isolates were significantly more resistant to trimethoprim/sulphamethoxazole, tetracycline, ciprofloxacin and gentamicin than the ones that are non ESBL producing (P<0.001).

#### Multi drug resistance

Multi-drug resistance was noted among 57% of *E*. *coli* and 82% of *K*. *pnuemoniae*. This is probably attributed to inappropriate use of these antibiotics in Uganda without guidance of culture results coupled with unavailability of functional microbiology laboratories in the country and unregulated over-the counter purchase of suboptimal doses. In Uganda, at least 40% of the patients who visit a health facility are treated with an antibiotic [4]. Nonexistence of policy on over-the-counter access to antibiotics is a big problem in Uganda. Due to unrestricted use of antimicrobials, participants could have taken antibiotics before coming to hospital and this has a direct influence on the above results since participants were recruited only basing on whether they had fever or diarrhea. While restricted access to antibiotics is important, the dilemma also comes in the need to balance it against the need to maintain access for the sizable proportion of the population that lacks access to doctors since lack of access to effective and affordable antibiotics still can lead to worse consequences.

#### Prevalence of ESBLs

ESBL production is a very vital mechanism of resistance among Enterobacteriaceae. In our study, ESBL production among *Escherichia coli* was 12%; lower than a similar study in Kampala-Uganda that reported 23% [23]. This is probably because more antibiotic use and abuse is likely to happen in the City than in the rural setting of Kasese where our study participants came from. The ESBLs prevalence among *E*. *coli* reported in our study is similar to that described in Tanzania of 15.5% [14]. There was higher isolation of ESBL among *K*. *pneumoniae* (23%) than that reported by other authors [14] [29] in Neighboring Tanzania and Kenya. Studies from other African countries show high ESBL rates of 33% (133/408) in Guinea Bissau [30] 34% (37/110) in Gabon [31] and 22% (54/244) in Madagascar [32]. A significant proportion of neonates in neighboring Tanzania, 25%(32/126) have been reported to carry ESBL with K. pneumoniae being the most predominant carrier [33]. In Niger [34], ESBL carriage among admitted children was 31% (17/55) and by the time of discharge, a further 15/16 had gained the carriage status. Whereas different studies employed different methods of detection of ESBLs, the results show a rising trend of ESBLs across the African continent.

Different ESBL prevalence rates have been recorded across Africa as generally high. Since there are few functional microbiology laboratories in most of the developing countries and medicines are dispensed without culture and resistance testing data, β-lactam antibiotics are likely to be glossily misused. Rising numbers of ESBLs in Uganda may have far reaching consequences and may mean that Uganda’s expenditure on effective antibiotics will rise if no effective mitigation interventions are put in place. Similar studies across the world have documented the predominance of ESBL producing *E*. *coli* and *K*. *pneumoniae* in the gastrointestinal tract and their potential to evolve into multiple drug resistant strains [32].

## Conclusion

We demonstrated high antimicrobial resistance, including multidrug resistance, among *E*. *coli* and *K*. *pneumoniae* isolates in pastoralist out-patients of rural Western Uganda (S1 Table).

## Recommendations

We recommend a study to link the high rates of drug resistance in humans to drug resistance in animals and environment in a One Health approach to establish the sources and drivers of this problem to inform public health. We also recommend molecular studies like whole genome sequencing to further characterize the resistance genes in these bacteria.

## Supporting information

S1 Table (DTA)

## Abbreviations

AMR: Antimicrobial resistance
CLSI: Clinical and laboratory standards institute
MDR: Multi-drug Resistance
ESBL: extended spectrum β-lactamase
EUCAST: European Committee on Antimicrobial Susceptibility Testing
WHO: World Health Organization
QEPA: Queen Elizabeth Protected Area
NDM-1: New Delhi Metallo-β-Lactamase-1
LMIC: Low and Middle Income Countries
CAP: College of American Pathologists
UNCST: Uganda National Council for Science and Technology

## References

[1] Laxminarayan R DA, Wattal C, Zaidi AK, Wertheim HF and Sumpradit N. Antibiotic resistance, the need for global solutions. The Lancet Infectious diseasesb 2013;13(12:1057–98). Pubmed Central PMCID: 24252483. Epub 2013/11/ 21. 10.1016/S1473-3099(13)70318-9 24252483

[2] Barker AK, Brown K, Ahsan M, Sengupta S and Safdar N. Social determinants of antibiotic misuse: a qualitative study of community members in Haryana, India. BMC public health. 2017 4 19;17(1):333 10.1186/s12889-017-4261-4 . Pubmed Central PMCID: 5395834. 28420365 PMC5395834

[3] Butaye P, Argudin, M.A. and Threlfall, J. Introduction to antimicrobial-resistant foodborne pathogens. In Antimicrobial Resistance and Food Safety Cambridge, MA, USA2015.

[4] Mukonzo JK, Namuwenge PM, Okure G, Mwesige B, Namusisi OK and Mukanga D. Over-the-counter sub-optimal dispensing of antibiotics in Uganda. Journal of multidisciplinary healthcare. 2013;6:303–10. 10.2147/JMDH.S49075 . Pubmed Central PMCID: 3753154. 23990728 PMC3753154

[5] De Kraker M DP, Grundmann H, BURDEN study group. Mortality and hospital stay associated with resistant Staphylococcus aureus and Escherichia coli bacteria: estimating the burden of antibiotic resistance in Europe. PLoS medicine. 2011;. 10.1371/journal.pmed.1001104 PMC3191157 22022233

[6] Centers for Disease Control. Antibiotic Resistance Threats in the United States. Controlhttp://wwwcdcgov/drugresistance/threat-report-. 2013.

[7] Allegranzi B NS, Combescure C, Graafmans W, Attar H, Donaldson L, Pittet D et al, Burden of endemic health-care-associated infection in developing countries: systematic review and meta-analysis. Lancet. 2011;377:228–41. 10.1016/S0140-6736(10)61458-4 21146207

[8] Poirel L, Revathi G, Bernabeu S, Nordmann P. Detection of NDM-1-producing Klebsiella pneumoniae in Kenya. Antimicrobial agents and chemotherapy. 2011 2;55(2):934–6. 10.1128/AAC.01247-10 . Pubmed Central PMCID: 3028766. 21115785 PMC3028766

[9] Stephen E Mshana, Mecky Isaac Matee and Mark Rwenyemamu. Antimicrobial resistance in human and animal pathogens in Zambia, Democratic Republic of Congo, Mozambique and Tanzania: an urgent need of a sustainable surveillance system. Annals of Clinical Microbiology and Antimicrobials 2013;12(28). 10.1186/1476-0711-12-28 PMC3852305 24119299

[10] Moremi N, Claus H, Mshana SE. Antimicrobial resistance pattern: a report of microbiological cultures at a tertiary hospital in Tanzania. BMC infectious diseases. 2016 12 13;16(1):756 10.1186/s12879-016-2082-1 . Pubmed Central PMCID: 5154146. 27964724 PMC5154146

[11] Kateete DP, Kabugo U, Baluku H, Nyakarahuka L, Kyobe S, Okee M, et al Prevalence and antimicrobial susceptibility patterns of bacteria from milkmen and cows with clinical mastitis in and around Kampala, Uganda. PloS one. 2013;8(5):e63413 10.1371/journal.pone.0063413 . Pubmed Central PMCID: 3646745. 23667611 PMC3646745

[12] Asiimwe BB, Baldan R, Trovato A, Cirillo DM. Prevalence and molecular characteristics of Staphylococcus aureus, including methicillin resistant strains, isolated from bulk can milk and raw milk products in pastoral communities of South-West Uganda. BMC infectious diseases. 2017 6 13;17(1):422 10.1186/s12879-017-2524-4 . Pubmed Central PMCID: 5470224. 28610560 PMC5470224

[13] Asiimwe BB, Baldan R, Trovato A, Cirillo DM. Molecular epidemiology of Panton-Valentine Leukocidin-positive community-acquired methicillin resistant Staphylococcus aureus isolates in pastoral communities of rural south western Uganda. BMC infectious diseases. 2017 1 05;17(1):24 10.1186/s12879-016-2124-8 . Pubmed Central PMCID: 5216539. 28056833 PMC5216539

[14] Mshana SE, Falgenhauer L, Mirambo MM, Mushi MF, Moremi N, Julius R, et al Predictors of blaCTX-M-15 in varieties of Escherichia coli genotypes from humans in community settings in Mwanza, Tanzania. BMC infectious diseases. 2016 4 29;16:187 10.1186/s12879-016-1527-x . Pubmed Central PMCID: 4850702. 27129719 PMC4850702

[15] Disassa N, Sibhat B, Mengistu S, Muktar Y, Belina D. Prevalence and Antimicrobial Susceptibility Pattern of E. coli O157:H7 Isolated from Traditionally Marketed Raw Cow Milk in and around Asosa Town, Western Ethiopia. Veterinary medicine international. 2017;2017:7581531 10.1155/2017/7581531 . Pubmed Central PMCID: 5337877. 28316862 PMC5337877

[16] Ursula Theuretzbacher CÅ, Stephan Harbarth. Linking sustainable use policies to novel economic incentives to stimulate antibiotic research and development Infectious disease reports. 2017 9(6836). 10.4081/idr.2017.6836 PMC5391537 28458797

[17] Critchlow R AJP, Driciru M., Rwetsiba A., Stokes E.J., Tumwesigye C., Wanyama F. et al Spatiotemporal trends of illegal activities from ranger-collected data in a Ugandan national park. Version of Record online. 2015 10.1111/cobi.12538 25996571

[18] BD PhoenixTM PID Panels. http://wwwbdcom/. 2013.

[19] Carroll KC, Glanz BD, Borek AP, Burger C, Bhally HS, Henciak S, et al Evaluation of the BD Phoenix automated microbiology system for identification and antimicrobial susceptibility testing of Enterobacteriaceae. Journal of clinical microbiology. 2006 10;44(10):3506–9. 10.1128/JCM.00994-06 . Pubmed Central PMCID: 1594749. 17021074 PMC1594749

[20] Falagas ME KP, Bliziotis IA. The diversity of definitions of multidrug resistant (MDR) and pandrug-resistant (PDR) Acinetobacter baumannii and Pseudomonas aeruginosa J Med Microbiol. 2006;55(12):1619–29. 17108263 10.1099/jmm.0.46747-0

[21] David Paterson. A step closer to extreme drug resistance (XDR) in gram-negative bacilli. Clin Infect Dis. 2007;45(9):1179–81. 10.1086/522287 17918079

[22] Kallen A, Hidron AI, Patel J, Srinivasan A. Multidrug resistance among gram-negative pathogens that caused healthcare-associated infections reported to the National Healthcare Safety Network 2006–2008. Infect Control Hosp Epidemiol. 2010;31(5):528–31. 10.1086/652152 20334552

[23] Najjuka CF, Kateete DP, Kajumbula HM, Joloba ML, Essack SY. Antimicrobial susceptibility profiles of Escherichia coli and Klebsiella pneumoniae isolated from outpatients in urban and rural districts of Uganda. BMC research notes. 2016 4 25;9:235 10.1186/s13104-016-2049-8 . Pubmed Central PMCID: 4843195. 27113038 PMC4843195

[24] Mwansa M. Songe BMHo, Knight-Jones Theodore J. D. and Delia Grace. Antimicrobial Resistant Enteropathogenic Escherichia coli and Salmonella spp. in Houseflies Infesting Fish in Food Markets in Zambia. Int J Environ Res Public Health 2017;14(21). 10.3390/ijerph14010021 PMC5295272 28036049

[25] Christine Ocokoru, Anguyo Robert, Philip Govule, Katongole Simon-Peter. Prevalence and Drug Susceptibility of Isolates of Urinary Tract Infections Among Febrile Under-Fives in Nsambya Hospital, Uganda. Open Science Journal of Clinical Medicine 2015;3(6):199–204.

[26] Chaula T, Seni J, Ng'walida N, Kajura A, Mirambo MM, DeVinney R, et al Urinary Tract Infections among HIV-Positive Pregnant Women in Mwanza City, Tanzania, Are High and Predicted by Low CD4+ Count. International journal of microbiology. 2017;2017:4042686 10.1155/2017/4042686 . Pubmed Central PMCID: 5307130. 28255302 PMC5307130

[27] Mshana SE, Kamugisha E, Mirambo M, Chakraborty T, Lyamuya EF. Prevalence of multiresistant gram-negative organisms in a tertiary hospital in Mwanza, Tanzania. BMC research notes. 2009 3 26;2:49 10.1186/1756-0500-2-49 . Pubmed Central PMCID: 2667529. 19323805 PMC2667529

[28] Blomberg B, Jureen R, Manji KP, Tamim BS, Mwakagile DS, Urassa WK, et al High rate of fatal cases of pediatric septicemia caused by gram-negative bacteria with extended-spectrum beta-lactamases in Dar es Salaam, Tanzania. Journal of clinical microbiology. 2005 2;43(2):745–9. 10.1128/JCM.43.2.745-749.2005 . Pubmed Central PMCID: 548071. 15695674 PMC548071

[29] Kiiru J KS, Goddeeris BM, Butaye P. Analysis of beta-lactamase phenotypes and carriage of selected beta-lactamase genes among Escherichia coli strains obtained from Kenyan patients during an 18-year period. BMC Microbiol. 2012;12(155). 10.1186/1471-2180-12-155 PMC3464591 22838634

[30] Isendahl J, Agata Turlej-Rogacka, Manjuba C, Rodrigues A, Giske CG, Naucler P. Fecal carriage of ESBL-producing E. coli and K. pneumoniae in children in Guinea-Bissau: a hospital-based cross-sectional study. PloS one. 2012;7(12). 10.1371/journal.pone.0051981 PMC3527401 23284838

[31] Schaumburg F, Alabi A, Kokou C, Grobusch MP, Kock R, Kaba H, et al High burden of extended-spectrum beta-lactamase-producing Enterobacteriaceae in Gabon. The Journal of antimicrobial chemotherapy. 2013 9;68(9):2140–3. 10.1093/jac/dkt164 . 23645586

[32] Andriatahina T, Frédérique Randrianirina, Hariniana ER, Talarmin A, Raobijaona H, Buisson Y, et al High prevalence of fecal carriage of extended-spectrum beta-lactamase-producing Escherichia coli and Klebsiella pneumoniae in a pediatric unit in Madagascar. BMC infectious diseases. 2010;10:204 10.1186/1471-2334-10-204 20624313 PMC2912907

[33] Nelson E, Kayega J., Seni J., Mushi M. F., Kidenya B. R., Hokororo, et al Evaluation of existence and transmission of extended spectrum beta lactamase producing bacteria from post-delivery women to neonates at Bugando Medical Center, Mwanza-Tanzania. BMC research notes. 2014 7(279). 10.1186/1756-0500-7-279 PMC4014626 24886506

[34] Woerther PL, Cécile Angebault, Jacquier H, Hugede HC, Janssens AC, Sayadi S, et al Massive increase, spread, and exchange of extended spectrum beta-lactamase-encoding genes among intestinal Enterobacteriaceae in hospitalized children with severe acute malnutrition in Niger. Clin Infect Dis 2011;53(7):677–85. 10.1093/cid/cir522 21890771

